# External Nasal Reconstruction in Patients With Extranodal Natural Killer (NK)/T-cell Lymphoma, Nasal Type

**DOI:** 10.7759/cureus.99730

**Published:** 2025-12-20

**Authors:** Misato Ueda, Tadashi Nomura, Shunsuke Sakakibara

**Affiliations:** 1 Department of Plastic Surgery, Kobe University Graduate School of Medicine, Kobe, JPN; 2 Department of Plastic Surgery, Hyogo Cancer Center, Akashi, JPN

**Keywords:** bone graft, cartilage graft, extranodal nk/t-cell lymphoma, nasal deformity, rhinoplasty surgery

## Abstract

Extranodal NK/T-cell lymphoma, nasal type (ENKTL-NT), is a rare and aggressive lymphoma of NK or T-cell origin, characterized by necrotizing and destructive granulomatous lesions that primarily affect facial midline structures. In the head and neck region, it often leads to extensive destruction of soft tissue, bone, and cartilage, potentially resulting in external nasal deformity and necrosis. This study reports on nasal reconstruction and its clinical course in patients with ENKTL-NT. Two patients with ENKTL-NT, who achieved remission following chemoradiotherapy, underwent external nasal reconstruction. Both cases involved autologous tissue reconstruction. Although external nasal morphology improved in both cases, long-term follow-up revealed contracture and secondary deformity, suggesting that outcomes were not entirely satisfactory. Accurate assessment of the remaining nasal components-including soft tissue and skeletal framework-is critical for selecting an appropriate reconstructive strategy.

## Introduction

Extranodal NK/T-cell lymphoma, nasal type (ENKTL-NT), is a malignancy associated with Epstein-Barr virus infection that was formally recognized by the World Health Organization in 2001. ENKTL-NT typically manifests as necrotizing lesions in the nasal cavity or upper aerodigestive tract, and more rarely, in extranasal sites such as skin or the gastrointestinal tract [1]. ENKTL-NT is characterized by necrotizing, destructive granulomatous lesions that predominantly affect the midface. Severe destruction of soft tissue, bone, and cartilage can result in external nasal deformity or necrosis. ENKTL-NT historically carried a poor prognosis; however, recent advances in clinical, pathological, genetic, and molecular characterization have led to new chemoradiotherapy regimens that significantly improve survival in early-stage disease [1,2]. Despite remission, facial deformities caused by ENKTL-NT can persist, imposing significant functional and psychological burdens. Reconstructive surgery in these patients is particularly challenging due to radiation-induced tissue damage, fibrosis, and soft tissue atrophy. We present two cases of external nasal deformity in patients with remission-stage ENKTL-NT treated with staged reconstructive surgery and discuss the technical considerations.

## Case presentation

Case 1

**Figure 1:**
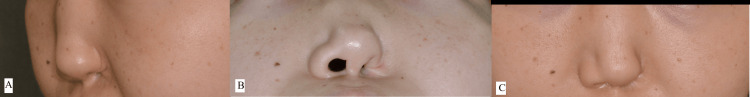
Preoperative clinical photographs in Case 1, showing left alar defect and nostril narrowing (A) Oblique view. (B) Inferior view. (C) Frontal view

**Figure 2:**
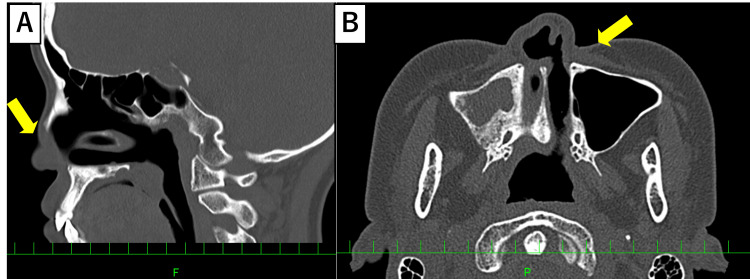
CT findings in Case 1, showing left alar and septal defect with cartilage thinning (yellow arrow) (A) Sagittal view. (B) Axial view

**Figure 3:**
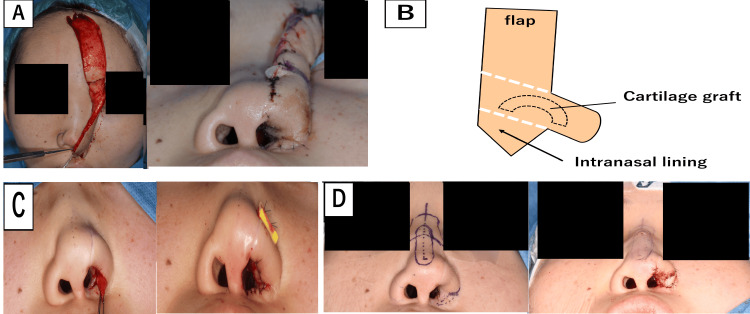
Surgical procedure in Case 1 (A) Findings and schematic of the first surgery. Reconstruction of the soft tissue defect of the nasal ala was performed using a forehead flap combined with an auricular cartilage graft to the ala. (B) Surgical illustration. (C) Second surgery. Refinement of the nasal alar contour was performed. (D) Third surgery. An iliac bone graft was placed on the nasal dorsum, and a composite graft from the auricle was applied to the alar base Image credit: Figure 3B is an original image created by the author Misato Ueda

**Figure 4:**
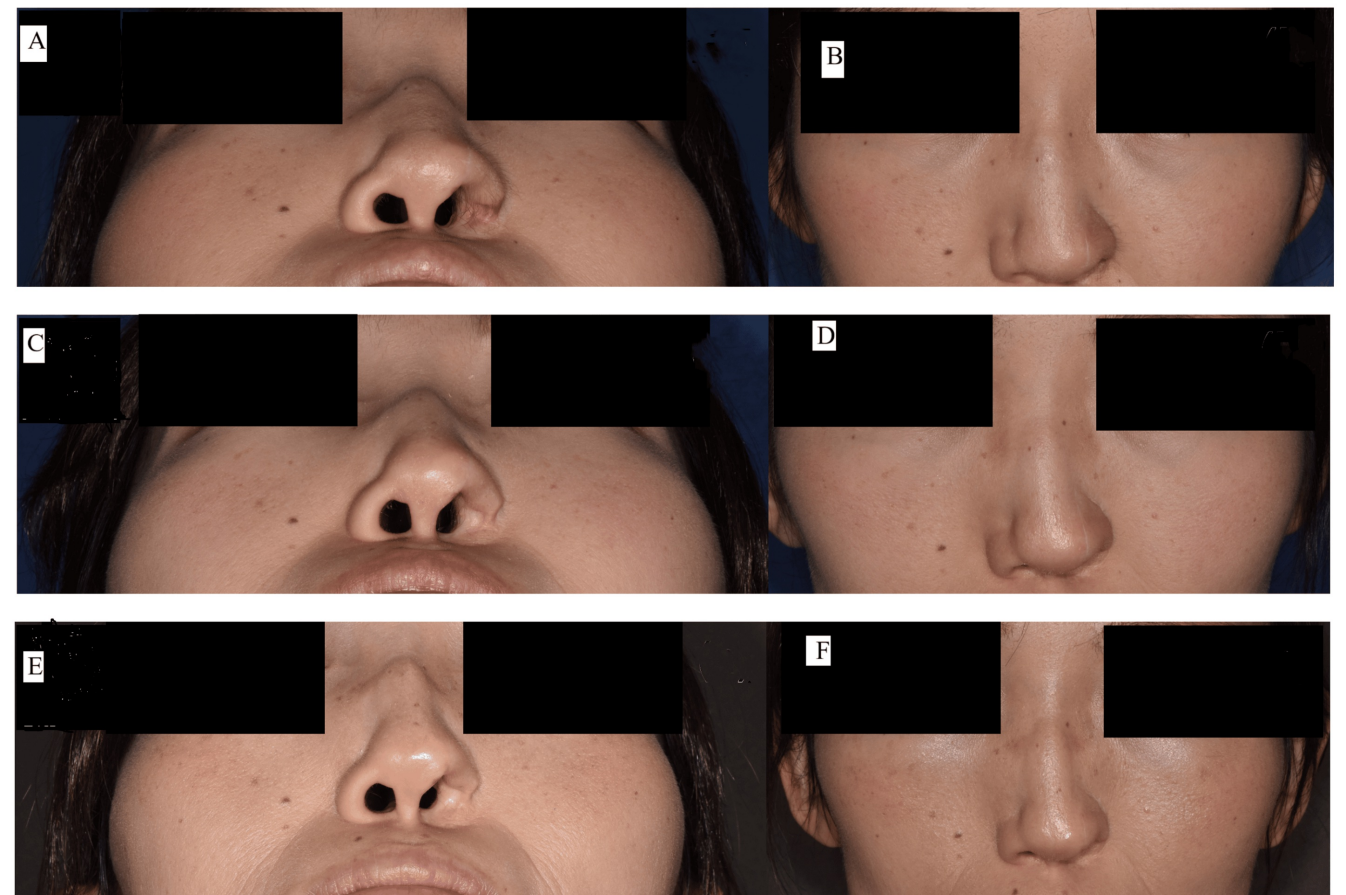
Postoperative views of Case 1 (A) Inferior and (B) frontal views three months postoperatively. (C) Inferior and (D) frontal views 10 months postoperatively. (E) Inferior and (F) frontal views seven years postoperatively, showing progressive soft tissue atrophy

A 24-year-old woman presented for external nasal reconstruction. She was diagnosed with ENKTL-NT two years prior and treated with chemoradiotherapy and hematopoietic stem cell transplantation, achieving remission. She exhibited a defect of the left nasal ala and adjacent soft tissue, along with significant narrowing of the left nostril and saddle nose deformity (Figure 1). CT imaging revealed a defect of the left ala and nasal septum, with thinning of the cartilage (Figure 2). A staged reconstructive approach was planned. Initially, the soft tissue defect of the left nasal ala was reconstructed using a forehead flap combined with an auricular cartilage graft to restore the alar structural framework. The left ala was incised to delineate areas of tissue deficiency, and the harvested auricular cartilage was sculpted to provide adequate structural support. The distal portion of the forehead flap was used as intranasal lining while simultaneously augmenting the alar subunit. Two weeks after the initial procedure, flap division was performed and the nasal contour refined. In the second stage, flap defatting and scar revision were carried out to optimize soft tissue aesthetics. In the third stage, a cantilever-shaped iliac bone graft was positioned on the nasal dorsum, and a composite graft harvested from the auricle was applied to further reconstruct the alar subunit (Figure 3). In total, three staged procedures were performed for nasal alar reconstruction. Although a significant improvement of the external nasal contour was achieved, progressive atrophy and deformity of the nasal ala recurred over time (Figure 4).

Case 2

**Figure 5:**
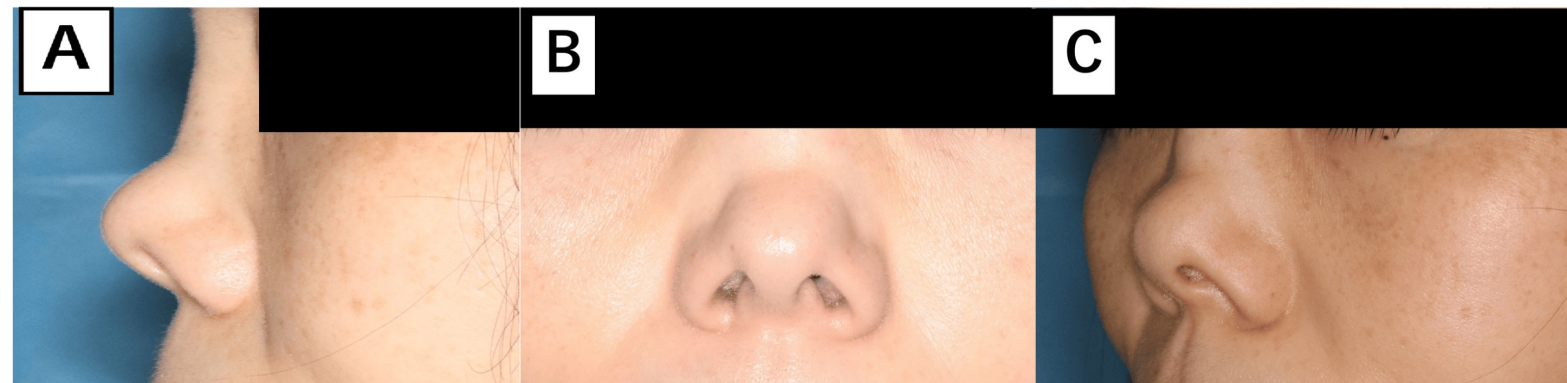
Preoperative clinical photographs in Case 2, showing saddle nose and short nose deformity (A) Lateral view. (B) Frontal view. (C) Oblique view

**Figure 6:**
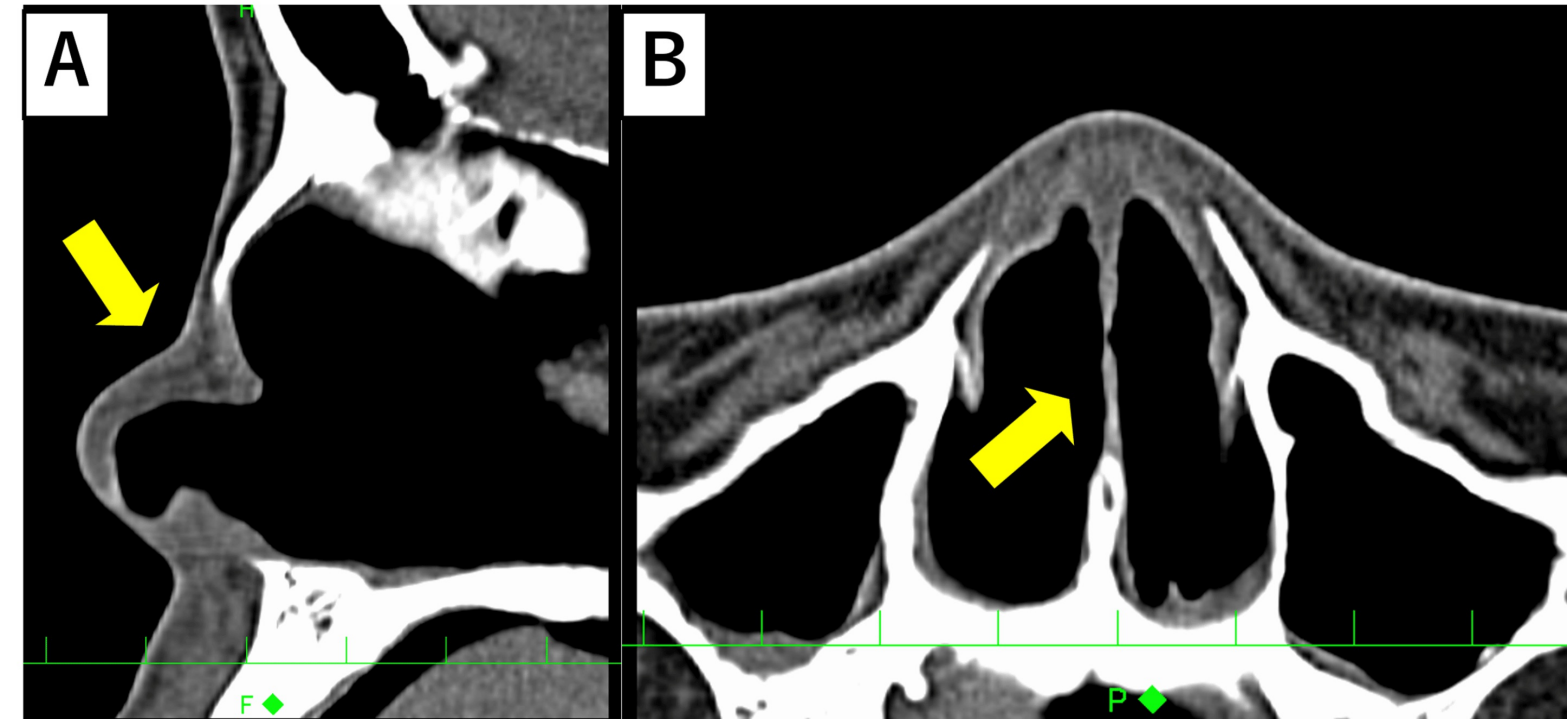
CT findings in Case 2, showing skin, mucosa, and cartilage thinning (yellow arrow) (A) Sagittal view. (B) Axial view

**Figure 7:**
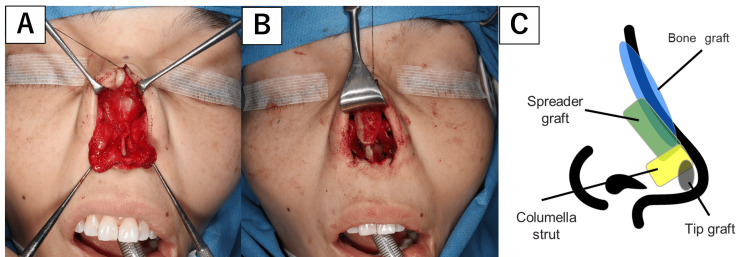
Surgical procedure in Case 2. (A) An open rhinoplasty approach was performed. The cartilage was thin and fragile. (B) The sculpted costal cartilage and iliac bone were grafted to restore the nasal contour. (C) Surgical illustration Image credit: Figure 7C is an original image created by the author Misato Ueda

**Figure 8:**
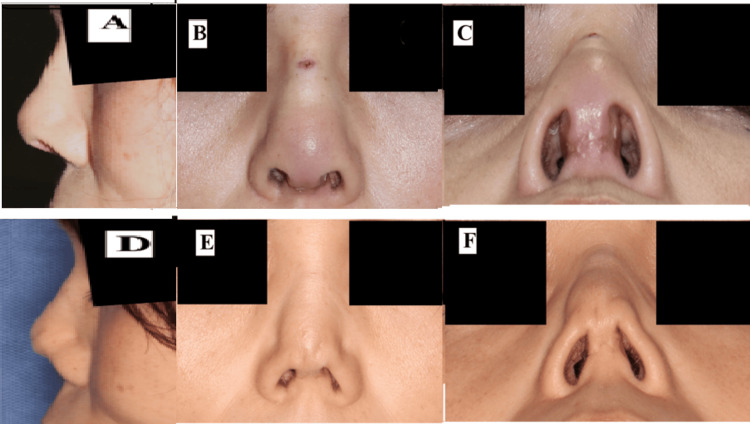
Postoperative views of Case 2 (A) Lateral, (B) frontal, and (C) inferior views two months postoperatively. (D) Lateral, (E) frontal, and (F) inferior views 10 months postoperatively, showing soft tissue atrophy and recurrent shortening

A 45-year-old woman, in remission from ENKTL-NT diagnosed six years earlier, was referred for nasal deformity. She exhibited a pronounced saddle nose and shortened nasal length (Figure 5). CT showed thinning of the skin, mucosa, and cartilage (Figure 6). As no soft tissue defect was present, reconstruction was planned using iliac and costal cartilage grafts alone. An open surgical approach was performed, and to address the deficiency of the structural support, harvested costal cartilage was fashioned into a columellar strut and spreader grafts, which were then sutured and secured to the remaining native cartilage. To correct the saddle-nose deformity, a cantilever-shaped iliac bone graft was placed on the nasal dorsum to restore its contour. While monitoring skin advancement, the fragmented costal cartilage was wrapped in processed autologous tissue and positioned to refine the nasal tip morphology (Figure 7). Over time, tissue atrophy and nasal retraction were observed, resulting in a more prominent short-nose appearance. Although the saddle-nose deformity showed improvement compared with the preoperative state, residual short-nose deformity remained, and the patient expressed a desire for additional treatment (Figure 8).

## Discussion

External nasal deformity in ENKTL-NT results from ischemic necrosis due to vascular destruction by tumor cells, as well as adverse effects of chemoradiotherapy [1,2]. The resultant tissue defects, fragility of structural components, and soft tissue atrophy contribute to surgical complexity. The specific pattern and extent of damage vary by case. Nasal deformity significantly impacts patients’ appearance, increasing psychosocial distress and reducing social function and emotional well-being [3]. Reconstruction is particularly challenging due to the effects of the primary disease and prior radiation. Only a few reports on nasal reconstruction for ENKTL-NT exist. Duong et al. [4] describe staged nasal reconstruction in such patients, emphasizing the complexity of defects and technical difficulties.

Effective nasal reconstruction requires consideration of the skin envelope, inner lining, and skeletal framework [5]. Rohrich et al. [6] proposed six fundamental principles for nasal reconstruction: 1) native tissue preservation whenever possible, 2）reconstruction of the defect rather than entire subunits, 3) prioritization of primary debulking, 4) use of axial-pattern flaps if possible, 5) minimization of staged surgeries, and 6) optimal contour as the aesthetic goal. In our cases, we prioritized native structure preservation and targeted reconstruction of the defect. However, due to thin and irradiated soft tissues and weakened supporting structures, progressive atrophy and contour deformities occurred over time. Late radiation effects such as fibrosis, vascular compromise, and poor tissue expansion capacity likely contributed to these outcomes [7,8]. Adequate vascularized tissue and stable structural support are essential for successful nasal reconstruction. In Case 1, the use of a well-vascularized flap, such as the radial forearm flap, for the intranasal lining, combined with the establishment of a firm central support prior to the forehead flap transfer, would have been more appropriate. In Case 2, additional soft tissue augmentation should have been considered to achieve a more favorable outcome.

Surgical planning must include an accurate assessment of residual tissues, accounting for irradiated skin stiffness and impaired blood flow. Preoperative assessment of vascular distribution is particularly useful in reconstructive surgery for the face after radiotherapy. Evaluations such as contrast-enhanced CT, angiography, and indocyanine green fluorescence imaging can provide valuable information regarding vascular distribution and perfusion, helping to optimize flap design, volume, and shape to minimize postoperative atrophy. Autologous tissues, such as dermofat grafts, cartilage (crushed or shaved), composite grafts, or vascularized flaps with auricular cartilage, may be considered for secondary corrections. However, the recipient bed must be evaluated for perfusion and mechanical support. Overcorrection may be necessary to compensate for future resorption.

Given these challenges, staged procedures and long-term follow-up are essential. As treatment outcomes for ENKTL-NT continue to improve, demand for nasal reconstruction may increase. Although some improvement in nasal morphology was achieved, persistent contracture and deformity over time limited the final outcome. Precise evaluation of each nasal component and individualized reconstructive planning are critical.

## Conclusions

Even after remission of ENKTL-NT, patients may have persistent external nasal deformity due to soft tissue atrophy and cartilage necrosis. Reconstruction must be tailored based on a detailed evaluation of missing or compromised tissue components. When soft tissue is deficient, flap reconstruction or tissue augmentation is required. When cartilaginous or bony support is lacking, rigid framework reconstruction is necessary. In both cases presented, autologous reconstruction was performed after chemoradiotherapy. Despite initial improvements, long-term deformity progression limited satisfactory outcomes. In patients with ENKTL-NT, such preoperative vascular assessments are also beneficial for planning reconstruction. Reconstruction in patients with ENKTL-NT must consider irradiated tissue characteristics, limited extensibility, and vascularity to minimize complications and achieve durable results.

## References

[1] Sánchez-Romero C, Bologna-Molina R, Paes de Almeida O, Santos-Silva AR, Prado-Ribeiro AC, Brandão TB, Carlos R (2021). Extranodal NK/T cell lymphoma, nasal type: an updated overview. Crit Rev Oncol Hematol.

[2] Al-Hakeem DA, Fedele S, Carlos R, Porter S (2007). Extranodal NK/T-cell lymphoma, nasal type. Oral Oncol.

[3] Kucur C, Kuduban O, Ozturk A (2016). Psychological evaluation of patients seeking rhinoplasty. Eurasian J Med.

[4] Duong CM, Ngo DQ, Tran TD, Ngo QX, Le QV (2019). Total nasal reconstruction for nasal defect after treatment for extranodal natural killer/T cell lymphoma, nasal-type: a case report. Int J Surg Case Rep.

[5] Losco L, Bolletta A, Pierazzi DM (2020). Reconstruction of the nose: management of nasal cutaneous defects according to aesthetic subunit and defect size. A review. Medicina (Kaunas).

[6] Rohrich RJ, Griffin JR, Ansari M, Beran SJ, Potter JK (2004). Nasal reconstruction--beyond aesthetic subunits: a 15-year review of 1334 cases. Plast Reconstr Surg.

[7] Borrelli MR, Shen AH, Lee GK, Momeni A, Longaker MT, Wan DC (2019). Radiation-induced skin fibrosis: pathogenesis, current treatment options, and emerging therapeutics. Ann Plast Surg.

[8] Olascoaga A, Vilar-Compte D, Poitevin-Chacón A, Contreras-Ruiz J (2008). Wound healing in radiated skin: pathophysiology and treatment options. Int Wound J.

